# Helium Plasma Radiofrequency Brow Lift: Description of a Novel Technique and Retrospective Study on Safety, Efficacy, and Durability

**DOI:** 10.1093/asjof/ojaf031

**Published:** 2025-04-30

**Authors:** Melinda Lacerna Kimbrell

## Abstract

**Background:**

Restoration of ideal eyebrow position and shape is paramount in facial aesthetics. Coagulation and contraction of the forehead tissue using subperiosteal application of thermal energy eliminates the need for tissue excision or fixation, permitting a closed, technically straightforward, nonendoscopic approach.

**Objectives:**

The aim of this study is to describe a novel brow-lift technique using subperiosteal application of thermal energy from a helium plasma radiofrequency (RF) device (Renuvion; Apyx Medical, Clearwater, FL).

**Methods:**

In this single-center, retrospective study, records for patients who underwent a brow lift with helium plasma RF were reviewed. Demographic, treatment, and safety information were collected. Efficacy and stability of the brow lift were measured by a blinded review of ≥90-day posttreatment photographs, changes in brow and forehead height, and patient satisfaction questionnaires.

**Results:**

Twenty-six patients were included in the analysis, with the longest follow-up being 3 years and 3 months. Of the 9 patients who had ≥90-day postoperative photographs, blinded reviewers correctly identified posttreatment images in 78% (7/9). The mean change in forehead height (lateral brow, brow arch, medial brow, and glabella to the hairline) decreased between 4.1 and 4.9 mm. The mean change in brow height (lateral brow, medial brow, and brow arch to the hairline to lash line) increased between 1.5 and 2.1 mm. Among 15 patients who completed the questionnaire, 87% (*n* = 13) of patients were satisfied/very satisfied with their results, and 73% (*n* = 11) would recommend treatment. There were no incidents of scar alopecia.

**Conclusions:**

This new technique for subperiosteal brow lifting using helium plasma RF may be safe, effective, and long-lasting. Further studies need to be conducted to confirm the findings in this paper.

**Level of Evidence: 4 (Therapeutic):**

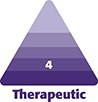

Restoration of ideal brow position is an integral part of facial rejuvenation. When performed effectively, brow-lift procedures can open up the periorbital area, resulting in a more youthful and refreshed look. Current techniques can be classified as nonsurgical or surgical. Nonsurgical techniques include the use of neurotoxins,^1^ fillers,^2^ threads, and radiofrequency (RF).^3^ Although these interventions are less technically challenging to perform and carry a lower risk of complications, adequate control of brow position is difficult, and results are temporary.^4^

Surgical brow lift has evolved significantly over the years, with techniques commonly classified as open or closed.^4^ Open techniques include coronal and smaller hairline incision techniques, as well as direct brow lifts.^5,6^ Although effective, these techniques are falling out of favor because of the risk of scarring, scar alopecia, and permanent scalp numbness. Transpalpebral techniques from the upper eyelid may circumvent these issues; however, they also are less likely to achieve and maintain the ideal brow position. Closed techniques, such as endoscopic brow lifts, are generally preferred by patients because of shorter operative times, less scarring, and a faster recovery.^7^ Although these are important advantages, tissue fixation (eg, unicortical screws, resorbable soft-tissue anchors, and threads^8,9^) makes them more complicated, time-consuming, and expensive, and carries the risk of suture granulomas, infection, extrusion, excess scarring, and alopecia of access sites.^10^ Irrespective of whether an open or closed technique is used, skin excision can cause scar alopecia in ∼2.8% of patients.^10^ In addition, endoscopic brow-lift techniques often produce an unwanted side effect of elongating or lengthening the forehead height, making this technique relatively contraindicated in patients with preexisting long foreheads. More recently, the gliding brow-lift technique has gained popularity;^11^ however, this technique includes the use of the hemostatic net, which can have a steep learning curve, prolong the surgical time, and produce hypertrophic scarring in certain patients.

Here, the author describes a novel brow-lifting technique that uses helium plasma RF (Renuvion, Apyx Medical, Clearwater, FL^12^) as an alternative to traditional surgical brow-lift procedures. Helium plasma RF is cleared by the United States FDA for the subcutaneous delivery of RF energy and/or helium plasma where coagulation/contraction of soft tissue is needed and, more specifically, for use in the neck and submental area to improve the appearance of lax skin and in the body for aesthetic body contouring.^12^ The brow-lifting technique described here relies on subperiosteal tissue coagulation and resultant contraction, producing brow elevation and forehead shortening, without tissue excision or fixation. To our knowledge, this is the first study to describe the subperiosteal application of this device.

## METHODS

### Study Design

This retrospective, single-center study reviewed medical records of patients aged 18 years and older who received helium-based plasma RF treatment in the subperiosteal layer of the forehead between March 2021 and September 2023. Those who underwent other surgical or nonsurgical brow procedures within 6 months before or after the brow lift were excluded. Patients could have additional procedures at the time of the brow lift. De-identified demographic, procedure and adverse event information were collected.

### Primary Outcomes

The study's key outcome measures evaluated the safety, efficacy, and durability of a novel subperiosteal brow-lifting technique using helium plasma RF. The primary efficacy measure was a masked, qualitative assessment of posttreatment photographs (≥90 days) compared with baseline, in which 3 blinded reviewers were asked to identify the posttreatment image. Independent photographic reviewers (Renuvion clinical educator nurses with experience in aesthetic and plastic surgery) were trained in the study protocol to select the posttreatment image (right or left, randomized) from each dual image set. They assessed eyebrow position, upper eyelid area, brow lines, wrinkles, brow laxity, and the overall brow and periorbital area appearance. Correct identification of the posttreatment image by at least 2 of 3 reviewers was considered a success. Among patients who consented to photographic assessment, photographs suitable for quantitative assessment were evaluated for changes in brow-to-hairline distance (forehead height) and brow to lash line distance (brow elevation in relation to the base of the upper eyelid). The lash line, a static structure not expected to move following surgical intervention, was used as a reference point to measure brow elevation relative to the upper eyelid. Patients who consented also completed a satisfaction survey, created by the authors and approved by the IRB, at their follow-up appointment at least 90 days postprocedure.

Adverse events (AEs) were evaluated, excluding expected treatment effects like edema and bruising and AEs related to concurrent procedures. Data were analyzed with descriptive statistics.

This study was approved by Sterling IRB (Atlanta, GA) and conducted in accordance with the 1964 Declaration of Helsinki and its amendments. Patients provided separate consent for photograph use and/or participation in the patient satisfaction portion of the study.

### Operative Technique

All procedures were performed in an accredited, office-based surgery center by the same board-certified plastic surgeon. A video of the procedure is provided (Video). The corrugators and procerus muscles were marked preoperatively with the patient animating in the holding area, using the same technique as for neurotoxin injections.

Three helium plasma RF handpieces were utilized in this study, selected based on patient anatomy, tissue thickness, and concurrent procedures. The Precise open handpiece (FDA cleared for both dermal resurfacing and subdermal use) was used when facial resurfacing was also planned. The dual-port APR handpiece was chosen for concurrent subdermal application in the neck, whereas the micro handpiece was selected with subdermal application to the lower eyelids.

The scalp, face, and neck areas were prepped with chlorhexidine and draped. Local anesthesia was injected into the anticipated scalp incisions (1% lidocaine with 1:100,000 epinephrine, 5 cc per incision site), and supraorbital and supratrochlear nerve blocks were performed (1% lidocaine with 1:100,000 epinephrine and 0.5% bupivacaine, 50/50 ratio, 4 cc per side). Next, tumescent solution (25 cc 1% lidocaine with 1:100,000 epinephrine, 500 mg tranexamic acid in 500 cc LR) was infiltrated into the forehead area in the subperiosteal plane for hydrodissection, with a total of 150 to 200 cc. A 25-G needle was injected into the forehead at a perpendicular angle; upon reaching the periosteum of the bone, it was withdrawn slightly (1 mm) before injecting the tumescent solution into the subperiosteal plane. This initiated the hydrodissection in the subperiosteal plane, forming small round blebs on the surface of the skin.

Two 1.5 cm paramedian scalp incisions were made (2 cm posterior to the hairline and 2 cm lateral to the forehead median). A subperiosteal dissection was extended caudally (to 0.5 cm below the brows) in a medial-to-lateral fashion to release the arcus marginalis brow attachments. The medial forehead dissection was carried out laterally, releasing the lateral temporal crest. Great care was taken laterally to avoid inadvertent injury to the superficial temporal artery, vein, and frontal branch of the facial nerve. Cephalic to the zygomatic arch, the frontal branch lies within an intermediate fascial layer, referred to as the “parotid temporal fascia,” which is deep to the temporoparietal fascia.^13^ It pierces the temporoparietal fascia near the sentinel vein before innervating the deep surface of the frontalis muscle. To prevent injury, the dissection was carefully maintained in the subperiosteal plane, which is deep to the frontalis muscle. This approach, described by Tabatabai and Spinelli in 2007,^14^ was performed without an endoscope, relying only on surface anatomy.

Once the arcus marginalis was released, the brows were elevated to the desired aesthetic position, and bilateral symmetry was confirmed. For asymmetry, tailored release of arcus marginalis, with further release on the side of the lower brow, was performed. After bilateral tailored release, symmetry was reassessed with the patient in an upright position.

The medial procerus and corrugator muscles were then partially avulsed using blunt surgical clamps to attenuate their brow depressor actions postoperatively. This was done by palpitating with the “smart” left hand (for right-handed surgeons) and the right hand performed the avulsion, relying on surface anatomy without an endoscope.

**Figure 1.**
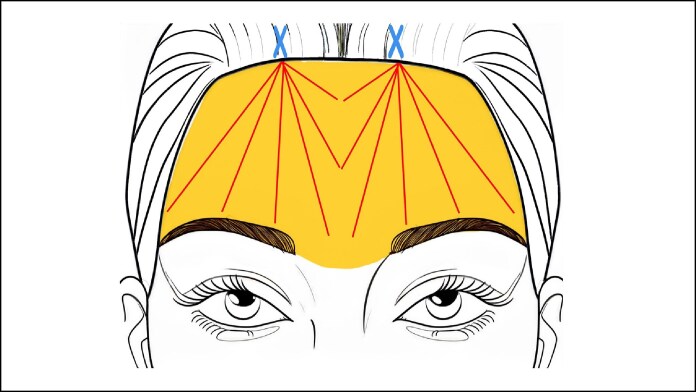
An X marks the areas of the 1.5 cm paramedian incisions and the areas highlighted depict the areas of subperiosteal dissection and application of helium plasma radiofrequency energy. Examples of treatment strokes used for each pass are shown in red. For patients in this study, ∼4 passes (each pass consisting of several strokes with 4 antegrade and retrograde strokes 2 cm apart at a speed of 1-2 cm/s) were performed per side.

Next, helium plasma RF energy was applied in the subperiosteal plane from the eyebrow base to the hairline in a medial-to-lateral fashion. Incisions and treatment area are shown in Figure 1. The resulting tissue contraction obviates the need for temporary or permanent fixation techniques. Each side received ∼4 passes (each consisting of 4 antegrade and retrograde strokes, 2 cm apart, at 1-2 cm/s). Proper technique is essential to prevent overheating, which can cause fibrosis or tissue damage. Maintaining a hand speed of 1 to 3 cm/s avoids excessive heating, with treatment limited to 4 passes per side and a total of 3 kJ per forehead side as an endpoint (6 kJ total), regardless of handpiece type. The Precise open/APR handpieces can reach energy settings of 80%, whereas the micro handpiece’s maximum energy setting is 30%. Energy was applied evenly to the lateral and medial brow. As the device tip nears the incision site, where treatment strokes converge, RF activation should be stopped to prevent localized overheating, with the device marker guiding the stopping point.

Following treatment, excess helium was evacuated using suction. After confirming hemostasis, the scalp incisions were closed with staples. Jackson–Pratt drains were not utilized.

Standardized postoperative instructions for brow lift, as well as for other concomitant procedures, were provided to patients. For the brow lift, a Coban compression wrap around the forehead area was applied for 24 h. Patients were advised to lie flat in a supine position whenever resting, with bed rest recommended for 24 to 48 h. At the Day 1 follow-up, the Coban wrap was removed and replaced with a facelift postoperative garment, to be worn continuously for 1 week, except when showering. No strenuous activity was permitted for 14 days postprocedure.

### Clinical Photography

At the beginning of the study period, pre- and postprocedure photography was performed with an iPad camera (Apple Inc., Cupertino, CA). In June 2023, a Canfield (Canfield Scientific, Parsippany, NJ) photography system was adopted for patient image capture. Emage PRO (Emage Medical, Charlotte, NC) software was utilized to measure the preoperative and postoperative measurements.

## RESULTS

### Study Demographics and Procedure Data

A total of 26 patients who underwent a minimally invasive brow lift with helium plasma were included in the analysis. Twenty-five patients were female, and 1 patient was male, with a mean age of 61 years (range, 39-74 years). Anesthesia types varied, including local anesthesia only (*n* = 2, 8%), intravenous sedation (most common; *n* = 17, 65%), and general anesthesia (*n* = 7, 27%). All patients underwent a concomitant procedure, with patients undergoing facelifts (73%), neck lifts (73%), facial fat grafting (77%), and helium plasma RF dermal resurfacing (46%). Average settings for the APR/Open Precise handpiece were 75% power (range, 30% to 80%), 1.6 L/min of helium flow (range, 1.2-2 L/min), and 4.8 kJ (range, 1.5-6 kJ). One patient was treated with the micro handpiece with the anterograde/retrograde technique, 30% power, 1 L/min helium flow, and 6 kJ total for the entire forehead. The total energy delivered was utilized as the endpoint, irrespective of the handpiece used. Demographic and procedure information is shown in Table 1.

**Table 1.** Patient Demographics and Treatment Characteristics

Demographics and treatment characteristics	Helium plasma RF brow lift (*n* = 26)
Sex, *n* (%)	
Male	1
Female	25
Age (years)	
Mean (min, max)	61 (39, 74)
Anesthesia type	
Local	2
Sedation	17
General	7
Other procedures (*n*, %)	
Facelift	19 (73)
Neck lift	19 (73)
Facial fat grafting	20 (77)
Plasma helium RF dermal resurfacing	12 (46)
Off-face procedures	6 (23)
Other	9 (35)

RF, radiofrequency; min, minimum; max, maximum.

### Treatment Effectiveness

**Figure 2.**
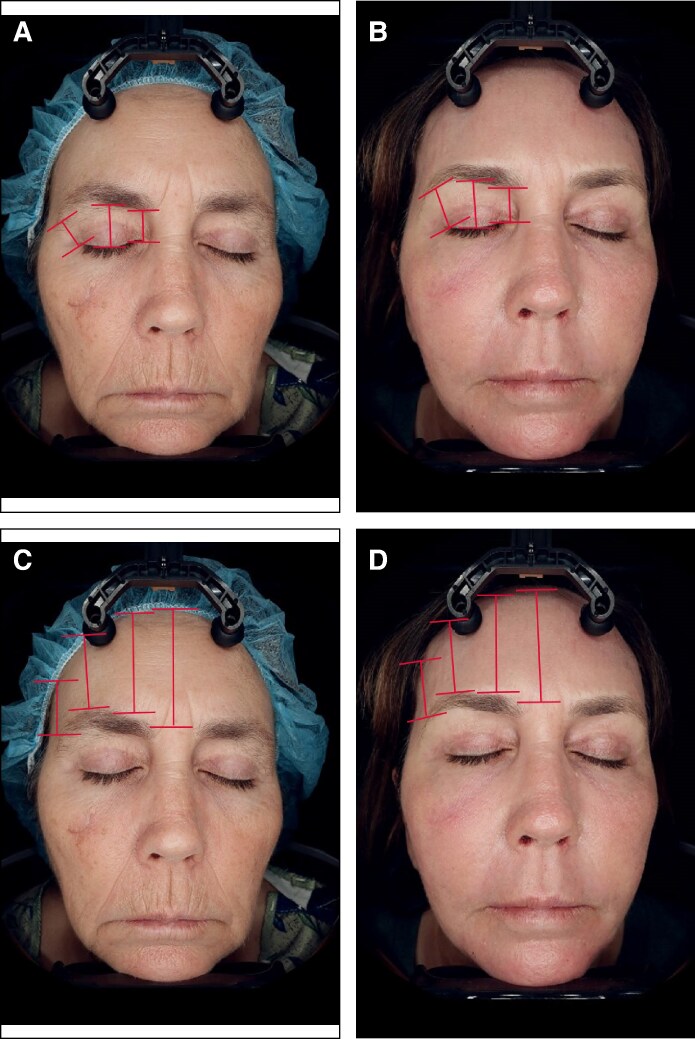
A 58-year-old patient at (A, C) baseline and (B, D) 100 days following treatment with subperiosteal helium plasma radiofrequency (RF) in the forehead along with a deep plane face and neck lift, platysmaplasty, fat grafting, and full-face helium plasma RF dermal resurfacing. Measures of forehead and brow elevation are shown. This patient experienced decreased distance from the hairline to the lateral brow (−2.11 mm), medial brow (−5.78 mm), glabella (−3.45 mm), and brow arch (−1.56 mm). The distance from the lash line to the lateral brow (2.69 mm), brow arch (1.25 mm), and medial brow (0.01 mm) all increased.

**Figure 3.**
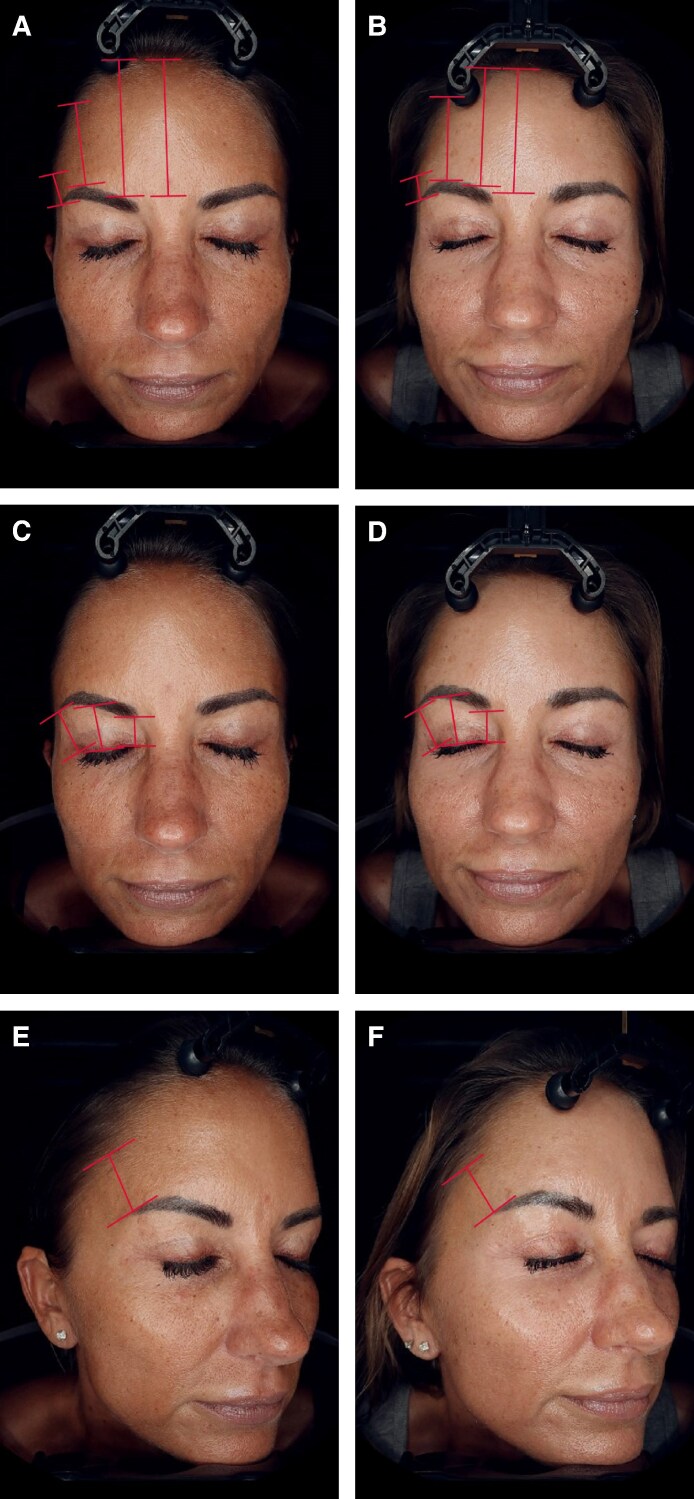
A 51-year-old patient at (A, C, E) baseline and (B, D, F) 11 months following treatment with subperiosteal helium plasma radiofrequency in the forehead only. Measures of forehead height and brow elevation are shown. This patient experienced decreased distance from the hairline to the lateral brow (−2.4 mm), medial brow (−10.03 mm), glabella (−7.29 mm), and brow arch (−5.12 mm). The distance from the lash line to the lateral brow (0.49 mm), brow arch (1.84 mm), and medial brow (0.38 mm) all increased.

**Figure 4.**
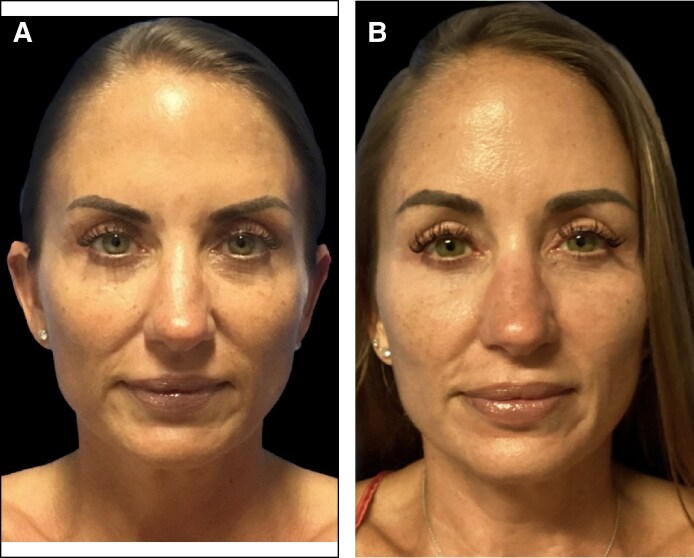
A 51-year-old female patient (A) before and (B) 11 months after a subperiosteal brow lift with helium radiofrequency plasma only.

**Figure 5.**
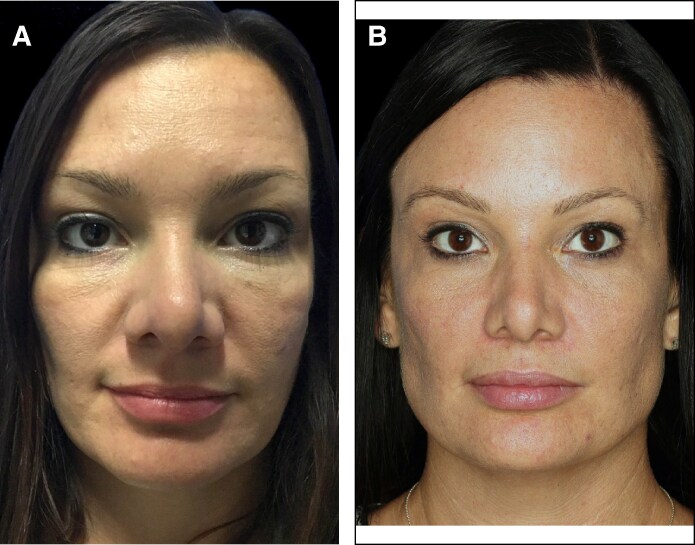
A 39-year-old female patient (A) before and (B) 3 years and 3 months after a subperiosteal brow lift with helium radiofrequency plasma only.

Of the 26 patients who were included in the analysis of procedure data, 14 agreed to participate in the photographic assessment. Of these 14 patients, 9 had follow-up photographs that were taken 90 days or more following the procedure, with a mean follow-up time for posttreatment photographs of 298 days (range, 128-765 days). The independent photographic review panel, consisting of 3 reviewers who required 2/3 agreement, correctly identified the treatment photograph for 78% of these patients. For 6/9 patients (67.7%), all 3 reviewers correctly identified the after image: for 1/9 patients (11.1%), 2 of the 3 reviewers correctly identified the after image, and for 2/9 patients (22.2%), 1 of the 3 reviewers correctly identified the after image. Examples of patient outcomes are presented in Figures 2-5.

Of the 14 consented patients who had follow-up photographs taken more than 90 days following the procedure, 6 had photographs of suitable quality for quantitative analysis. For these patients, the average time postprocedure was 216.8 days (range, 100-312 days). Measurements were taken from the lateral brow, medial brow, and brow arch to the hairline. The average distance between the lateral brow and the hairline decreased by 4.1 mm (standard deviation [SD], 3.6), the distance between the medial brow and the hairline decreased by 4.9 mm (SD, 3.6), and the distance between the brow arch and the hairline decreased by 4.1 mm (SD, 2.6), together indicating that patient forehead height had decreased, rather than increased. The distance from the glabella to the hairline also decreased by an average of 3.6 mm (SD, 2.2). Measurements from the brow arch and the medial and lateral brow to the lash line had increased. The average distance between the lateral brow and the lash line increased by 2.1 mm (SD, 1.7), the distance between the brow arch and the lash line increased by 1.8 mm (SD, 1.1), and the distance between the medial brow and the lash line increased by 1.5 mm (SD, 1.6). Patient measurements are shown in Table 2 and are indicated on Figures 1 and 2. Among the 6 patients with photographs of suitable quality for quantitative analysis, concomitant procedures included facelifts (67%), neck lifts (67%), facial fat grafting (67%), helium plasma RF dermal resurfacing (67%), and off-face procedures (33%).

**Table 2.** Changes in Forehead Height and Brow Elevation

Distance measured	Change in position, mm (*n* = 6)
*Changes in forehead height*	
Lateral brow to hairline (mm)	
Mean (SD)	−4.1 (3.6)
Minimum, maximum	−1.08, −10.68
Medial brow to hairline (mm)	
Mean (SD)	−4.9 (3.6)
Minimum, maximum	−0.39, −10.03
Brow arch to hairline (mm)	
Mean (SD)	−4.1 (2.6)
Minimum, maximum	−0.31, −6.53
Glabella to hairline (mm)	
Mean (SD)	−3.6 (2.2)
Minimum, maximum	−0.77, −7.29
*Changes in brow elevation*	
Lateral brow-to-lash line (mm)	
Mean (SD)	2.1 (1.7)
Minimum, maximum	0.49, 5.19
Medial brow to lash line (mm)	
Mean (SD)	1.5 (1.6)
Minimum, maximum	0, 3.47
Brow arch to lash line (mm)	
Mean (SD)	1.8 (1.1)
Minimum, maximum	0.73, 3.85

SD, standard deviation.

### Patient Satisfaction

A total of 14 patients agreed to complete the patient satisfaction questionnaire. Mean time to follow-up for the patient satisfaction survey was 364 days postprocedure (range, 106-944 days). A single patient provided 2 ratings, 1 for each side of the face, raising the number of responses in the analysis to 15. Overall, 87% (*n* = 13) of patients were satisfied with the results of their brow procedure, including 53% who were very satisfied and 73% (*n* = 11) who would recommend it to their friends and family. The most frequently noted improvements were “My upper face looks better” (60%, *n* = 9), “I feel like I look younger” (60%, *n* = 9), “My eyes seem more open” (60%, *n* = 9), and “My eyebrows seem elevated” (60%, *n* = 9; Table 3).

**Table 3.** Patient Satisfaction Questionnaire Results

Question/characteristic	Result, *n* (%) (*N* = 15)
Improvement in the area that was treated with helium-based plasma radiofrequency (Renuvion)?	
Yes	13 (87)
I feel like I look younger	9 (60)
My eyebrows seem elevated	9 (60)
My eyes seem more open	9 (60)
My upper face looks better	9 (60)
Improvement in wrinkles	8 (53)
Smoother skin texture	8 (53)
I feel like I look more refreshed	7 (47)
My skin looks brighter	7 (47)
Less sagging skin	6 (40)
My upper eyelid feels less heavy	6 (40)
My crow's feet seem improved	5 (33)
More even skin tone (color)	2 (13)
No	2 (13)
Satisfaction with the treatment	
Very satisfied	8 (53)
Satisfied	4 (27)
Slightly satisfied	1 (7)
Dissatisfied	1 (7)
Slightly dissatisfied	0 (0)
Very dissatisfied	1 (7)
Would you recommend the procedure to friends and family?	
Yes	11 (73)
No	4 (27)

### Safety Outcomes

AEs were collected for all 26 patients in the analysis. Most patients (*n* = 23, 88%) did not experience an AE. Two patients (8%) reported that their brow had dropped unilaterally, and 1 (3.8%) had facial nerve palsy. For the first patient, the left medial brow dropped following late formation of a seroma at 8 weeks postprocedure, which disrupted the adherence of the new, intended brow position. This patient required a surgical revision at 1 year postoperative, and now has restored and stable symmetry at 24 weeks postrevision. The second case of brow drop was attributed to CN VII neuropraxia and resolved spontaneously within 2 weeks. The patient with facial nerve palsy had undergone a concurrent high-SMAS facelift and developed neuropraxia of both the right frontal and zygomaticomaxillary branches of the right facial nerve. At 1 year postprocedure, the patient had complete return of facial movement and brow symmetry. No cases of scar alopecia, skin burns, skin necrosis, infection, hematoma, or permanent numbness in the supraorbital or supratrochlear distribution were reported.

## DISCUSSION

Taken together, these results suggest that brow lifting with helium plasma RF is a viable option for surgical brow lifting. Both blinded review and patient satisfaction data support efficacy, with 78% of blinded reviewers correctly selecting patients’ after images and 87% of the patients reporting high satisfaction with the results. All measures of postoperative forehead height show decreased values (Table 2), attributable to the soft-tissue contraction resulting from the application of helium plasma RF. This is an advantage over endoscopic techniques, which can elongate the forehead. Patients with long forehead heights are usually offered an excisional coronal brow lift; however, the nature of the incision and associated risk of scars that can traverse across the frontal hairline, along with longer recovery and permanent frontal scalp numbness (because of the transection of branches of the supraorbital or supratrochlear nerves), make this a highly undesirable option for most patients. Utilizing helium plasma RF to contract the forehead tissue instead of skin excision, which can result in considerable scarring, is a significant benefit. In addition to brow lifting, this procedure can theoretically be utilized in forehead reduction techniques to lower frontal hairlines that have receded from alopecia.

The author prefers helium plasma RF to the subperiosteal plane over the subgaleal plane as it is a fairly avascular, safer plane, situated deep to key anatomical structure, namely the supraorbital and supratrochlear neurovascular bundles, the frontal branch of the facial nerve, and the branches of the superficial temporal artery and vein tributaries. Furthermore, this technique obviates the need for hemostatic net sutures, which are used for subgaleal and subcutaneous techniques, such as the popular gliding brow lift.^11^ Hemostatic net sutures have a steep learning curve for surgeons, added suture cost, increase operative times, and may cause scarring for patients with higher Fitzpatrick skin types. Furthermore, the more superficial dissections required for subgaleal techniques and subcutaneous techniques can result in visible skin and soft-tissue irregularities that may take a while to settle, causing additional stress to the patient and the surgeon. In contrast, subperiosteal techniques, by their nature, are deeper dissections that do not produce surface irregularities.

This study is the first documentation of soft-tissue contraction in the subperiosteal plane produced by helium plasma RF. Previous animal studies by Holcomb and Schucker showed epidermal and dermal tissue contraction following application of helium plasma RF;^15^ however, no widely recognized studies or established techniques use RF in the subperiosteal plane for brow lifting, as evidenced by its absence in recent reviews.^16-18^ Ukoha et al. discussed the use of a fractionated RF needle device for brow lifting, with 1 patient achieving a 2.5 mm brow elevation and another achieving a 2 mm brow elevation at 6 months posttreatment.^3^

The results presented here demonstrate brow elevation laterally, medially, and at the brow arch relative to the upper lash line. The greatest elevation was observed laterally (the intended aesthetic effect), with a mean change in elevation of 2.1 mm. The medial brow remained relatively stable at 1.5 mm mean elevation, 0.3 mm lower than the lateral brow, avoiding the unintended “surprised” look. The brows are elevated without excessive lift in any 1 area, permitting a balanced aesthetic outcome. Brow lifting results with helium plasma RF are comparable with those reported with other methods. The authors of 1 study showed a mean brow lift at the lateral canthus of 1.85 mm, compared with 2.1 mm observed here, and 1.07 mm at the medial limbus, compared with 1.5 mm in this study.^19^ These values are consistent with those reported in other retrospective analyses of endoscopic techniques.^7^ Furthermore, the longevity of our subperiosteal brow-lift technique with helium plasma RF appears promising, with the longest follow-up of 3 years and 3 months, approaching the 5 year outcomes reported in endoscopic studies.^20^

The safety profile of helium plasma RF brow lift is also favorable, with results indicating it can be safely combined with other facial procedures, such as blepharoplasties, face and neck lifts, fat grafting, and helium plasma dermal resurfacing. Among 26 patients, 2 reported brow dropping—1 because of CN VII neuropraxia, which resolved spontaneously in 2 weeks, and another requiring revision after a late seroma. Additionally, 1 patient experienced transient frontal branch palsy following a concurrent facelift, resolving within a year. These complications also occur after endoscopic and other surgical techniques;^10^ however, because of the small number of patients in our analysis, it is difficult to make comparisons between the rates of these complications.

A key advantage of helium plasma RF is that it eliminates the need for fixation techniques, thereby reducing costs and the risks of palpability, extrusion, infection, and displacement associated with brow tissue fixation devices. Tissue contraction occurs during the procedure, eliminating the need for temporary or permanent fixation. Helium plasma RF also obviates the need for tissue excision in the scalp; therefore, the tension-free posthairline scalp incisions (1.5 cm each) can heal rapidly with minimal scarring and scar alopecia. Because the incisions are tension free, they require only skin stapler closure.

The device rapidly heats tissue to nearly 85°C, achieving coagulation within 0.04 s.^21-23^ This limits the increase of surface temperatures and allows for rapid cooling following coagulation, protecting the surface skin and surrounding tissue from inadvertent burns. Notably, no incidents of skin burns or necrosis were reported. Despite its favorable safety profile, this technique should be performed only by an experienced surgeon familiar with RF technology.

Although the helium plasma RF technique is a closed technique, an endoscope is not required. Instead, the surgeon relies on the forehead surface anatomy and landmarks, similar to marking toxin injection points. The arcus marginalis is released medially and laterally until the brows achieve adequate mobility. Once adequate mobility is sufficient, the brows are “pulled up” to the desired aesthetic brow position with the best symmetry possible. The final brow position is controlled by the surgeon's release, and the helium plasma RF energy is used to “fixate” this new elevated position by forehead tissue contraction. Although many surgeons perform the forehead release without endoscopic visualization, blind muscle avulsion theoretically increases the risk of bleeding. To avoid this risk, the procerus and corrugator muscles are infiltrated with tumescent solution containing epinephrine and tranexamic acid for at least 25 min before avulsion, ensuring proper vasoconstriction before initiating the brow-lift procedure. Additionally, the helium plasma RF device aids in coagulation, similar to electrocautery. No bleeding complications were observed in this study.

In addition to their established safety and efficacy,^14^ nonendoscopic limited-incision subperiosteal brow lifts with surface visualization offer several practical advantages. Endoscopic techniques have a steep learning curve, require expensive and cumbersome endoscopes, and can suffer from unclear intraoperative visualization and image fogging, even in the hands of the most experienced surgeons. Furthermore, procedures performed without an endoscope are 20 min shorter, on average.^14^ In the author's experience, the total surgical time for this technique is ∼30 min. The procedure can be performed under local anesthesia, reducing the need for anesthesia personnel, medications, and equipment. Although the cost of the helium plasma RF device itself is higher than an endoscope, the helium plasma RF device is arguably more versatile given that it can be used for a range of clinical applications, including skin tightening with liposuction and facial skin resurfacing.

The limitations of this study include the retrospective design and the small patient number. In addition, qualitative analysis was carried out over a wide range of follow-up periods, with a mean follow-up of 216.8 days (range, 100-312 days). In addition, inconsistencies in patient image capture make millimeter-level assessments challenging, highlighting the need for a larger study with standardized photography. The imaging system was upgraded during the study period, substantially improving consistency. Although the photographic reviewers were blinded, they were employees of the study sponsor: future studies could include crowdsourcing to reduce the potential for any bias. A limitation of the tested device is the lack of real-time visualization of tissue contraction or temperature monitoring, making it difficult to confirm whether target tissue reaches the optimal temperature for collagen contraction without overheating. However, the extremely brief duration required for coagulation suggests it likely occurs near the end of the probe as it moves through tissues. Because many patients underwent concomitant procedures, even with blinded review, improvements from other procedures could have influenced image identification. However, because these procedures were performed in different facial areas and tissue planes, brow position is unlikely to change unless zones of adherence, such as the arcus marginalis, are selectively released, given their dense adherence to the supraorbital bone.

Although the follow-up for patients included in the quantitative analysis was substantial, studies with follow-up beyond 5 years will better inform surgeons and patients about long-term outcomes. Because more patients undergo this procedure, larger retrospective studies that include several hundred patients, similar to those published for endoscopic brow lift, will help establish its safety and efficacy profile. Specifically, assessing changes in sensation or dysesthesia in a larger patient group would be valuable.

## CONCLUSIONS

This study indicates that subperiosteal brow lift with helium plasma RF may be a safe, effective, and durable option for surgical brow lifting. The technique elevates brow position both medially and laterally, with potentially long-lasting results. Requiring only two 1.5 cm incisions, it is technically straightforward and eliminates the need for an endoscope. The procedure time is short, and the absence of fixation eliminates the risk of palpability, extrusion, infection, displacement, and the need for revisional surgery to address these issues. Because patient demand grows for minimally invasive techniques with minimal downtime and lasting results, this technique may become an important option for surgical brow lifting.
